# A case-control study of Clostridioides difficile symptomatic infections in a pediatric cancer hospital

**DOI:** 10.1590/1984-0462/2023/41/2022117

**Published:** 2023-03-13

**Authors:** Adriana Maria Paixão de Sousa da Silva, Lara de Castro Barbosa, Leticia Maria Acioli Marques, Letícia Yasuda Carreira, Fernanda Maria Casimiro da Fonseca, Ana Paula Cordeiro Lima, Janaína Joice Martins Sodré, Luara Teofilo Pignati, Orlei Ribeiro Araújo, Dafne Cardoso Bourguignon da Silva, Fabianne Altruda de Moraes Costa Carlesse

**Affiliations:** a Instituto de Oncologia Pediátrica, São Paulo, SP, Brazil.; b Universidade Federal de São Paulo, São Paulo, SP, Brazil.; c Hospital Alemão Oswaldo Cruz, São Paulo, SP, Brazil.; d Instituto Adolfo Lutz, São Paulo, SP, Brazil.; e Universidade de São Paulo, São Paulo, SP, Brazil.

**Keywords:** Child, Clostridioides difficile, Cancer, Criança, Clostridioides difficile, Câncer

## Abstract

**Objective:**

The aim of this study was to analyze and identify documented infections and possible risk factors for *Clostridioides difficile* infections in children with cancer.

**Methods:**

This is a retrospective case-control study, carried out in a pediatric cancer hospital, covering the years 2016–2019. Matching was performed by age and underlying disease, and for each case, the number of controls varied from 1 to 3. Logistic regression models were used to assess risk factors.

**Results:**

We analyzed 63 cases of documented infection by *C. difficile* and 125 controls. Diarrhea was present in all cases, accompanied by fever higher than 38°C in 52.4% of the patients. Mortality was similar among cases (n=4; 6.3%) and controls (n=6; 4.8%; p=0.7). In all, 71% of patients in the case group and 53% in the control group received broad-spectrum antibiotics prior to the infection. For previous use of vancomycin, the *Odds Ratio* for *C. difficile* infection was 5.4 (95% confidence interval [95%CI] 2.3–12.5); for meropenem, 4.41 (95%CI 2.1–9.2); and for cefepime, 2.6 (95%CI 1.3–5.1). For the antineoplastic agents, the *Odds Ratio* for carboplatin was 2.7 (95%CI 1.2–6.2), melphalan 9.04 (95%CI 1.9–42.3), busulfan 16.7 (95%CI 2.1–134.9), and asparaginase 8.97 (95%CI 1.9–42.9).

**Conclusions:**

*C. difficile sympt*omatic infection in children with cancer was associated with previous hospitalization and the use of common antibiotics in cancer patients, such as vancomycin, meropenem, and cefepime, in the last 3 months. Chemotherapy drugs, such as carboplatin, melphalan, busulfan, and asparaginase, were also risk factors.

## RESUMO

**Objetivo:**

Analisar e identificar infecções documentadas e possíveis fatores de risco para infecções por *Clostridioides difficile* em crianças com câncer.

**Métodos:**

Estudo retrospectivo caso-controle em um hospital pediátrico oncológico, que abrangeu os anos de 2016–2019. O pareamento foi realizado por idade e doença de base e, para cada caso, o número de controles variou de um a três. Modelos de regressão logística foram utilizados para avaliar os fatores de risco.

**Resultados:**

Analisamos 63 casos de infecção documentados por *C. difficile* e 125 controles. A diarreia esteve presente em todos os casos, acompanhada de febre acima de 38°C em 52,4% dos pacientes. A mortalidade foi semelhante entre casos (n=4, 6,3%) e controles (n=6, 4,8%; p=0,7). No grupo caso, 71% dos pacientes e, no grupo controle, 53% deles receberam antibióticos de amplo espectro antes da infecção. Para uso prévio de vancomicina, a *Odds Ratio* para infecção por *C. difficile* foi de 5,4 (intervalo de confiança [IC95%] 2,3–12,5); para meropenem, 4,41 (IC95% 2,1–9,2) e, para cefepima, 2,6 (IC95% 1,3–5,1). Para os agentes antineoplásicos, a razão de chances para carboplatina foi de 2,7 (IC95% 1,2–6,2), para melfalano de 9,04 (IC95% 1,9-42,3), para bussulfano de 16,7 (IC95% 2,1–134,9) e, para asparaginase, de 8,97 (IC95% 1,9–42,9).

**Conclusões:**

A infecção sintomática por *C. difficile* em crianças com câncer associou-se à internação prévia e ao uso de antibióticos como vancomicina, meropenem e cefepime nos últimos três meses. Os quimioterápicos carboplatina, melfalano, bussulfano e asparaginase também foram fatores de risco.

## INTRODUCTION

*Clostridium difficile*, reclassified in 2016 to the new genus *Clostridioides*, is a spore-forming anaerobic Gram-positive bacillus, acquired from the environment or by fecal-oral route. It can colonize the gastrointestinal tract, especially in children younger than 2 years old.^1^ This is the most common cause of antimicrobial-associated diarrhea, and it is a frequent healthcare-related infection.^2^ Clinical symptoms can vary from asymptomatic colonization to pseudomembranous colitis with bloody diarrhea, fever, and intense abdominal pain. The disease may be severe, leading to bowel perforation, toxic megacolon, and death. The pathogenic strains are able to produce toxins A and B, which are responsible for the clinical manifestations by disrupting the cytoskeletal structure of the intestinal cells.^3^

Despite the increasing number of *C. difficile* infections (CDI) and their severity, CDI continues to be an underestimated cause of diarrhea in patients <18 years of age. One reason for the underestimation of CDI in children is the high rate of asymptomatic colonization (in infants 14–70%; in children 1–2 years of age, approximately 6%), followed by a common perception that young children are not susceptible to CDI. However, data indicate that this perception is valid only for neonates. In all other groups of children, the number of CDI continues to grow. A relatively large amount of data exists regarding prominent pediatric patients burdened with a high risk of CDI development, including Hirschsprung’s disease, inflammatory bowel disease, malignancies, hematological disorders, and immunodeficiency.^1^

Children with cancer are more than 15 times more likely to have the disease than healthy children. This increased susceptibility is caused by factors such as increased health care contact, immunosuppression caused by chemotherapy, and repeated and/or prolonged exposure to broad-spectrum antibiotics.^4^ Hospitalized children with cancer account for 15–25% of CDI in pediatric cohorts.^5,6^ Surveillance testing in pediatric oncology patients identified stool colonization in 29% of patients without gastrointestinal symptoms and in 55% of patients with prior CDI.^7^

The aims of this study were to identify potential risk factors related to the occurrence of CDI in children with cancer, assisted in a Brazilian pediatric oncology center, and describe the related complications and mortality.

## METHOD

This is a retrospective case-control study, in which all medical records of included patients, inpatients and outpatients, were reviewed, covering the period from January 2016 to January 2020. As cases, we included all patients with cancer and/or undergoing hematopoietic stem cell transplantation (HSCT), aged 0–18 years, with a documented infection by *C. difficile* (a test positive for toxins A and B, a positive glutamate dehydrogenase test, or a positive polymerase chain reaction for *C. difficile*) and clinical symptoms: fever (T>37.8°C), diarrhea with or without mucus or blood, abdominal pain, and no evidence of another infectious cause. There was no isolation of *C. difficile* in stool cultures.

For each case, at least one and at most three controls were included, using patients without CDI seen in the same period of diagnosis of the case. Matching was performed based on the type of cancer, age, and whether HSCT was performed. Variables studied were age, sex, underlying disease, HSCT, chemotherapy received in the last three months, antibiotic therapy in the last three months, and symptoms such as fever, diarrhea (number of episodes, duration, and presence of mucus and/or blood), abdominal pain, and vomiting.

Statistical analysis: Absolute and relative frequencies and summary measures (mean, median, minimum, maximum, and standard deviation) were calculated as indicated. Correlations between variables and the outcome CDI were evaluated via bivariate conditional logistic regression. Differences between frequencies were estimated using the chi-square test or Fisher’s exact test. For comparison of means, the T-test was used. We established a significance level of 5%. Tests were performed with IBM SPSS version 20.0 (Armonk, NY: IBM Corp.).

The study was approved by the Ethics Committee of Universidade Federal de São Paulo (UNIFESP) (protocol number 37966920.1.0000.5505, approved on 12/14/2020).

## RESULTS

There were 6583 hospitalizations in the period, with 63 patients with documented CDI (prevalence of 0.92%). In all, 22 cases occurred in patients under 5 years of age, 14 in the range of 5–10 years of age, and 27 between 10 and 18 years of age. Also, 125 controls were included. Demographic characteristics are shown in Table 1.

**Table 1.** Demographic and diagnostic data of patients by groups.

	Controls (n=125)	Cases (n=63)	p-value
Female	58 (46.4)	31 (49.2)	NS
Age (years, mean, SD)	9.1 (5.8)	8.9 (5.9)	NS
HSCT (n, %)	26 (20.8)	8 (12.7)	NS
Underlying disease (n, %)			
Central nervous system tumor	32 (25.6)	18 (28.6)	NS
Other solid tumors	26 (20.8)	12 (19.0)	NS
Non-Hodgkin lymphoma	9 (7.2)	5 (7.9)	NS
Acute myeloid leukemia	8 (6.4)	5 (7.9)	NS
Acute lymphocytic leukemia	35 (28)	11 (17.5)	NS
Hodgkin’s lymphoma	4 (3.2)	3 (4.8)	NS
Histiocytosis	4 (3.2)	3 (4.8)	NS
Retinoblastoma	4 (3.2)	1 (1.6)	NS
Myelodysplastic syndrome	3 (2.4)	3 (4.8)	NS
All-cause mortality in 30 days	4 (6.3)	6 (4.8)	NS

Differences between frequencies were estimated using the chi-square test or Fisher’s exact test. For comparison of means, the Student’s t-test was used. SD: standard deviation; NS: not significant; HSCT: hematopoietic stem cell transplantation.

Diarrhea was a symptom present in all cases, followed by abdominal pain (57%), hypotension (28.6%), and intestinal bleeding in only eight cases (12.7%). Oral metronidazole was the first-line treatment in 73% of cases, followed by intravenous metronidazole in 20.6%. In four (6.8%) cases, it was necessary to escalate therapy from metronidazole to vancomycin. Clinical and laboratory characteristics are described in Table 2.

**Table 2.** Clinical and laboratory data.

	Cases (n=63)
C-reactive protein (mean, SD)	100.8 (108.4)
Toxins A and B, n (%)	
Negative	4 (6.3)
Positive	59 (93.7)
Abdominal pain (n, %)	36 (57.1)
Fever, n (%)	33 (52.4)
Days to defervescence (median, range)	2 (1–11)
Diarrhea (n, %)	63 (100)
Days of diarrhea (median, range)	5 (1–41)
Enterorrhagia (n, %)	8 (12.7)
Days of enterorrhagia (median, range)	2.5 (1–21)
Hypotension n (%)	18 (28.6)
PICU admission (n, %)	23 (36.5)
PICU length of stay (median, range)	14.5 (3–41)
First-line antibiotic (n, %)	
Intravenous metronidazole	13 (20.6)
Oral metronidazole	46 (73)
Days of first-line antibiotic (median, range)	13.0 (1–36)
Second-line antibiotic	
Oral vancomycin	4 (6.3)
Days of second-line antibiotic (median, range)	23.5 (8–33)

PICU: Pediatric Intensive Care Unit; SD: standard deviation.

A positive test for toxins A and B was present in 59 (93.7%) of 63 cases. In the remaining cases, the diagnosis was made by polymerase chain reaction (two cases) and by positive glutamate dehydrogenase in two cases. Glutamate dehydrogenase was performed in only seven patients, being positive in all. In 17 cases, imaging tests were performed, such as ultrasound (three cases, 4.8%) and computed tomography (14, 22%). In these, increased thickness of intestinal wall was observed in 13 patients (76.5%).

Table 3 shows the binary logistic regression models for the various risk factors assessed for the outcome CDI. The variables included previous hospitalization, the use of the antibiotics cefepime, meropenem, and vancomycin in the last three months. The chemotherapy drugs melphalan, busulfan, carboplatin, and asparaginase were shown to increase the probabilities of this outcome (Table 4).

**Table 3.** Binary logistic regression models for the outcome “*C. difficile* infection”.

	Controls (n=125)	Cases (n=63)	*Odds Ratio* (95%CI)	p-value
Previous hospitalization in the last three months	73 (58.4)	51 (81.0)	4.74 (1.91–11.76)	0.001
Graft-versus-host disease, n (%)	6 (4.8)	6 (9.5)		NS
Neutropenia, n (%)	38 (30.4)	24 (38.1)	1.54 (0.78–3.02)	NS
Mucositis, n (%)	11 (8.8)	6 (9.5)	1.06 (0.35–3.21)	NS
Use of antibiotics in the last 3 months:				NS
Amikacin, n (%)	28 (22.4)	11 (17.5)	0.80 (0.37–1.72)	NS
Ceftriaxone, n (%)	45 (36.0)	25 (39.7)	1.11 (0.61–2.01)	NS
Cefepime, n (%)	54 (43.2)	40 (63.5)	2.61 (1.34–5.1)	0.005
Meropenem, n (%)	26 (20.8)	33 (52.4)	4.41 (2.11–9.19)	<0.001
Metronidazole, n (%)	17 (13.6)	5 (7.9)	0.60 (0.21–1.73)	NS
Vancomycin, n (%)	44 (35.2)	42 (66.7)	5.41 (2.33–12.59)	<0.001
Polymyxin B, n (%)	2 (1.6)	4 (6.3)	6.61 (0.72–60.86)	NS

95%CI: 95% confidence interval; NS: not significant.

**Table 4.** Binary logistic regression models for the outcome “*C. difficile* infection” according to antineoplastic agents used in the last three months.

	Controls (n=125)	Cases (n=63)	*Odds Ratio* (95%CI)	p-value
Cyclophosphamide	40 (32.0)	29 (46.0)	1.77 (0.96–3.27)	NS
Ifophosphamide	18 (14.4)	14 (22.2)	1.87 (0.83–4.20)	NS
Melphalan	4 (3.2)	10 (15.9)	9.04 (1.93–42.35)	0.005
Busulfan	1 (0.8)	8 (12.7)	16.75 (2.08–134.96)	0.008
Thiotepa	4 (3.2)	6 (9.5)	3.23 (0.78–13.35)	NS
Temozolomide	5 (4.0)	3 (4.8)	1.11 (0.24–5.20)	NS
Mercatopurine	20 (16.0)	10 (15.9)	1.02 (0.40–2.63)	NS
Thioguanine	7 (5.6)	8 (12.7)	2.89 (0.92–9.13)	NS
Cladribine	1 (0.8)	3 (4.8)	5.81 (0.59–57.47)	NS
Fludarabine	5 (4.0)	7 (11.1)	2.70 (0.84–8.62)	NS
Nelarabine	1 (0.8)	1 (1.6)	1.73 (0.10–30.76)	NS
Cytarabine	25 (20.0)	14 (22.2)	1.34 (0.59–3.04)	NS
Gemcitabine	1 (0.8)	2 (3.2)	4.61 (0.41–51.31)	NS
Azacitidine	5 (4.0)	2 (3.2)	0.80 (0.15–4.19)	NS
Hydroxyurea	2 (1.6)	1 (1.6)	0.78 (0.07–8.88)	NS
Vinblastine	19 (15.2)	9 (14.3)	0.98 (0.40–2.41)	NS
Vincristine	40 (32.0)	20 (31.7)	1.15 (0.6–2.22)	NS
Vinorelbine	2 (1.6)	1 (1.6)	1.000 (0.09–11.03)	NS
Etoposide	24 (19.2)	7 (11.1)	0.54 (0.22–1.34)	NS
Docetaxel	1 (0.8)	2 (3.2)	4.61 (0.41–51.31)	NS
Daunorubicin	11 (8.8)	6 (9.5)	1.18 (0.39–3.58)	NS
Doxorubicin	24 (19.2)	16 (25.4)	1.35 (0.66–2.75)	NS
Idarubicin	3 (2.4)	2 (3.2)	1.16 (0.18–7.33)	NS
Dactinomycin	1 (0.8)	4 (6.3)	6.27 (0.69–57.17)	NS
Cisplatin	15 (12.0)	13 (20.6)	2.33 (0.92–5.90)	NS
Carboplatin	16 (12.8)	16 (25.4)	2.67 (1.16–6.18)	0.022
Rituximab	1 (0.8)	4 (6.3)	8.00 (0.89–71.58)	NS
Bevacizumab	2 (1.6)	1 (1.6)	1.14 (0.10–12.66)	NS
Dasatinib	4 (3.2)	2 (3.2)	0.88 (0.16–4.89)	NS
Topotecan	2 (1.6)	5 (7.9)	4.08 (0.77–21.65)	NS
Tretinoin	1 (0.8)	3 (4.8)	6.69 (0.69–64.78)	NS
Irinotecan	2 (1.6)	3 (4.8)	2.64 (0.43–16.02)	NS
Asparaginase	2 (1.6)	8 (12.7)	8.97 (1.87–42.96)	0.006
Pegylated asparaginase	8 (6.4)	4 (6.3)	1.17 (0.31–4.35)	NS

NS: not significant.

## DISCUSSION

We observed in this study that the antibiotic most likely to be correlated with CDI was vancomycin, followed by meropenem and cefepime. Importantly, our data show that exposure in the last three months persists as a risk. Children with cancer are often exposed to broad-spectrum antibiotics, and in our study, 71% of patients in the case group and 53% in the control group received broad-spectrum antibiotics. Antibiotics are a well-known risk for the development of CDI. Different classes of antibiotics present different risks, with clindamycin, fluoroquinolones, cephalosporins, monobactams, and carbapenems presenting the greatest risks, according to the meta-analysis by Brown et al.^8^ Another important fact is that previous studies revealed that there may be a differential risk of CDI among the antipseudomonal β-lactam antibiotics used in patients during febrile neutropenia, with cefepime posing a greater risk than antipseudomonal penicillins.^4^

Our patients had fewer clinical signs than in general pediatric studies, with a lower percentage of fever and abdominal pain, for example. Although we expected a higher rate of treatment failures, only four cases required an escalation from metronidazole to vancomycin, which is quite similar to the rates reported in children without cancer.^1^

We observed also that exposure in the last 3 months to antineoplastic agents, such as carboplatin, melphalan, busulfan, and asparaginase, was linked to higher probabilities of CDI. Antineoplastic drugs can cause CDI without the concomitant use of antibiotics. The mechanism by which chemotherapeutics increase the risk of CDI is unclear, but it has been proposed that they may act as antibiotics and cause changes in the intestinal flora. In addition, they can directly damage the mucosa and decrease the ability of intestinal mucosa cells to regenerate and repair.^9^

Due to increased use of chemotherapy regimens with damage to the gastrointestinal tract (e.g., methotrexate, 5-fluorouracil, irinotecan, topotecan, etoposide, cisplatin, carboplatin, paclitaxel, docetaxel, melphalan, busulfan, cyclophosphamide, and ifosfamide), associated with a greater use of antibiotics, prolonged immunosuppression, and longer hospital stays, previous studies have reported that CDI is 2.5 times more common in patients with hematological malignancies than in those with solid tumors, and 1.4 times higher in HSCT recipients than in other cancer patients.^9–11^ We did not find these greater risks in our study, probably due to the low strength of the case and control design. This may also explain the fact that we did not find any difference in mortality between groups. CDI has been linked to increased overall mortality,^10^ but it is difficult to quantify its direct attributable effect. It can also indirectly contribute to higher mortality by affecting nutritional status, requiring more invasive procedures, or delaying chemotherapy.^12^

There are other obvious weaknesses in the study design. We tried to match controls that had similar diagnoses and hospitalization dates, but it is uncertain that this match is ideal or representative because there is enormous heterogeneity among these children, even for the same diagnosis, with large variations involving immunological and genetic characteristics. Thus, some degree of bias in the determination of *Odds Ratios* cannot be ruled out. To the best of our knowledge, this is the first study to evaluate the risks and outcomes of CDI in Brazilian children with cancer.

CDI in children with cancer was related to previous hospitalization and the use of antibiotics commonly used in cancer patients, such as vancomycin, meropenem, and cefepime. Chemotherapeutic drugs, such as carboplatin, melphalan, busulfan, and asparaginase, were also related to higher risk of this infection.
